# Prevalence of motor impairment in residents of New South Wales, Australia aged 55 years and over: cross-sectional survey of the 45 and Up cohort

**DOI:** 10.1186/s12889-020-09443-5

**Published:** 2020-09-04

**Authors:** R. D. Herbert, J. L. Taylor, S. R. Lord, S. C. Gandevia

**Affiliations:** 1 Neuroscience Research Australia (NeuRA), Barker St, Randwick, NSW 2031 Australia; 2 University of New South Wales, Sydney, Australia; 3 Edith Cowan University, Perth, Australia

**Keywords:** Motor impairment, Weakness, Fatigue, Contracture, Balance, Coordination

## Abstract

**Background:**

The population prevalence of many diseases is known. However, little is known of the population prevalence of motor impairments.

**Methods:**

The aim of this study was to determine the point prevalence of specific motor impairments (weakness, fatigue, contracture, impaired balance and impaired coordination) in the population aged 55 years and older resident in New South Wales, Australia in 2018. 55,210 members of the 45 and Up cohort were invited to participate in a follow-up survey that included questions on motor impairment. Responses were received from 20,141 people (36%). Calibrated estimates of prevalence of specific motor impairments, and of having at least one motor impairment, were obtained using survey weights based on the known multivariate distributions of age, gender and geographical location (28 regions) in the population.

**Results:**

More than one-third of adults aged over 55 residing in New South Wales have difficulty using their hands, arms or legs. The prevalence of each motor impairment (muscle weakness, fatigue, contracture, impaired balance or impaired coordination) in this population is between 4 and 12%. The prevalence of at least one of these impairments is 21%. The prevalence of at least one impairment in people aged 85 and over is 42%. Women consistently had more difficulty using hands, arms and legs, and more motor impairment, than men. Difficulty using hands, arms and legs and the prevalence of all motor impairments, especially poor balance, greatly increased with age.

**Conclusion:**

The prevalence of specific motor impairments in older Australian adults is high - comparable to that of the most prevalent diseases. There may be merit in considering motor impairment as a significant public health problem in its own right.

## Background

Studies of disease prevalence contribute to quantification of disease burden [1]. The current study is concerned with the prevalence of motor impairments instead of the prevalence of disease.

Motor impairments interfere with motor ability. This study focuses on five motor impairments: muscle weakness, fatigue, contracture, impaired balance and impaired coordination. Fatigue is a complex motor impairment because it includes both a reduction in the capacity of a muscle to produce force during sustained motor tasks and an increased sense of effort, exhaustion or weariness [2]. Impaired balance is also complex because it can reflect peripheral failure or failure of multisensory integration and high level executive functions [3].

Motor impairments can have many causes. For example muscle weakness may be caused by stroke, neuromuscular disease or prolonged bed rest. Nonetheless, motor impairments with diverse causes can share common mechanisms, have the same consequences, contribute to the same clinical syndromes and respond to the same interventions. So, to use the same example, muscle weakness may be mediated by muscle atrophy, impair ambulation, increase risk of falling and respond to exercise therapy, regardless of whether the weakness is caused by stroke, neuromuscular disease or bed rest. To the extent that motor impairments share common mechanisms, have the same consequences and respond to the same interventions it may be useful to study motor impairments, not just the diseases or injuries that cause motor impairments.

While there have been many disease-specific studies of the prevalence of motor impairment (e.g. prevalence of fatigue in multiple sclerosis [4]), there have been relatively few population-based studies. Notable exceptions include studies of population prevalence of weakness in Brasil [5], USA [6–8] and Ecuador [9]; fatigue in Norway [10], England [11] and USA [12]; and impaired balance and coordination in Brasil [13], Norway [14], Colombia [15] and the USA [16]. Nonetheless, with the possible exception of estimates of the prevalence of weakness, estimates of the prevalence of specific motor impairments are sparse. There do not appear to be any data on the population prevalence of contracture [17].

As there have been relatively few population-based studies of the prevalence of motor impairment and, to our knowledge, no population-based studies of the prevalence of specific motor impairments in the Australian population, we sought to determine the prevalence of weakness, fatigue, contracture, impaired balance and impaired coordination in residents of the state of New South Wales aged 55 years and older in 2018.

## Methods

The 45 and Up cohort was assembled between 2006 and 2009 by sending invitations to New South Wales residents aged 45 and over who were randomly sampled from the Department of Human Services (Medicare Australia) enrolment database [18]. The database includes people in aged-care facilities. Medicare is the system that provides free or subsidised health services to all Australian citizens and residents, as well as some non-residents. Invitations were sent by mail. By design, there was oversampling of residents in rural areas and people aged over 80. All people living in remote areas were invited to participate. The response rate was approximately 18%. People who had not been invited to participate could request to participate in the study but only 0.1% of the cohort was recruited in this way. Recruitment continued until December 2009 when there were 267,153 participants in the cohort. At the time of entry into the study, participants provided extensive demographic and health data. Data were self-reported or provided by a proxy (e.g., family member or carer).

There have been two further waves of date collection from this cohort. The second of these is currently underway: in each of the years 2018–2021, invitations to provide follow-up data have been or will be sent to approximately one-quarter of those who are alive and contactable and who have not asked to withdraw from the study. At 1 January 2018, 89% of the cohort was alive. In 2018, invitations were sent to 55,210 participants. This paper summarises the data that 20,141 participants (36% of those invited) provided in 2018 by mail or email in response to those invitations.

In the 2018 follow-up, participants were asked to respond to questions about motor impairment. Specifically, they were asked:

“In general, how would you rate your balance?” Response options were “poor”, “fair”, “good”, “very good” and “excellent”. (In the subsequent analyses, participants were assumed to have impaired balance if they rated their balance as poor.)

“Do you have any difficulty using your hands, arms or legs to carry out everyday activities?” If the response was “yes”, additional response options for each of “hands”, “arms” and “legs” were “neither”, “one” and “both”.

“If you have difficulty, is the difficulty due to any of the following?” Response options, for each of “weakness”, “muscles tire easily – fatigue”, “limited joint movement from contracture” and “poor coordination – clumsiness” were “hand/s”, “arm/s” and leg/s”.

The target population for this study was the 2,215,637 people residing in New South Wales aged 55 years and over in 2018. To estimate prevalence in the target population as accurately as possible, study participants were assigned sampling weights based on three control variables: age, sex and geographical location. Geographical location was categorised using the 28-level 2011 Statistical area level 4 (SA4) code [19]. Sampling weights were calculated using the multivariate distributions of the control variables in the population from the 2015 Australian Statistical Geography Standard (ASGS) survey conducted by the Australian Bureau of Statistics. (2015 was the last year in which the Bureau used 2011 SA4 codes.) Sampling weights for each of 224 cells (4 age categories × 2 sexes × 28 geographical areas) were obtained by dividing the count in the population by the count in the sample. The weights were scaled up by a factor of 1.063 to account for the population growth in New South Wales from 2015 to 2018 [20]. Crude estimates of prevalence were adjusted by the sampling weights using ‘pweight’s in Stata 15.1.

## Results

The mean sampling weight for the 20,141 participants who provided follow-up data was 115, indicating that on average, each survey participant represented 115 people in the target population. After truncating survey weights at a maximum of 500 (necessary for just 0.2% of the cohort) the coefficient of variation of the survey weights was 0.61, indicating that the distribution of age, sex and geographical location in the sample was moderately representative of the age, sex and geographical location of the population. Most of the 20,141 participants provided complete responses. As a typical example, 19,277 participants (96%) provided data on ability to use hands, arms or legs.

A large proportion of the target population (37.1%, 95% CI 36.3 to 37.9%) reported difficulty using hands, arms or legs. Table 1 shows the distribution of difficulty using hands, arms and legs by age and sex.

**Table 1:** Estimated prevalence (%) of difficulty using hands, arms or legs, by age and sex

Age	Men (***N*** = 1,055,316)	Women (*N* = 1,160,321)	All (***N*** = 2,215,637)
**Difficulty using hands, arms or legs**			
55–64 (*N* = 930,520)	30.9 (28.9 to 33)	35.8 (34.1 to 37.5)	33.4 (32.1 to 34.7)
65–74 (*N* = 719,450)	32.7 (31 to 34.4)	37.1 (35.6 to 38.6)	34.9 (33.8 to 36.1)
75–84 (*N* = 393,247)	36.5 (33.8 to 39.2)	45.1 (42.6 to 47.6)	41.1 (39.3 to 43)
85+ (*N* = 172,420)	52.1 (47.6 to 56.7)	59.6 (55.1 to 63.9)	56.8 (53.6 to 60)
Total (55+) (*N* = 2,215,637)	33.7 (32.5 to 34.9)	40.1 (39.1 to 41.2)	37.1 (36.3 to 37.9)
**Difficulty using hands**			
55–64 (*N* = 930,520)	9.5 (8.3 to 10.9)	14.2 (13 to 15.5)	11.8 (11 to 12.7)
65–74 (*N* = 719,450)	10 (8.9 to 11.1)	14.4 (13.3 to 15.5)	12.1 (11.4 to 12.9)
75–84 (*N* = 393,247)	10.3 (8.7 to 12.2)	16.8 (15 to 18.7)	13.3 (12.1 to 14.6)
85+ (*N* = 172,420)	17.5 (14.3 to 21.2)	25.2 (21.5 to 29.4)	20.3 (17.8 to 23.1)
Total (55+) (*N* = 2,215,637)	10.6 (9.8 to 11.4)	15.3 (14.6 to 16.1)	12.8 (12.3 to 13.4)
**Difficulty using arms**			
55–64 (*N* = 930,520)	8.2 (7.1 to 9.4)	9.2 (8.2 to 10.2)	8.7 (7.9 to 9.5)
65–74 (*N* = 719,450)	7.8 (6.9 to 8.8)	9.3 (8.4 to 10.3)	8.6 (7.9 to 9.3)
75–84 (*N* = 393,247)	9 (7.4 to 10.8)	11.9 (10.3 to 13.6)	10.3 (9.2 to 11.6)
85+ (*N* = 172,420)	13.2 (10.4 to 16.7)	19.1 (15.7 to 23)	15.3 (13.1 to 17.9)
Total (55+) (*N* = 2,215,637)	8.7 (8 to 9.4)	10.3 (9.6 to 10.9)	9.4 (9 to 9.9)
**Difficulty using legs**			
55–64 (*N* = 930,520)	14.5 (13 to 16.1)	15.5 (14.2 to 16.8)	15 (14 to 16)
65–74 (*N* = 719,450)	16.1 (14.8 to 17.5)	17.7 (16.5 to 18.9)	16.9 (16 to 17.8)
75–84 (*N* = 393,247)	19.8 (17.6 to 22.2)	26.4 (24.2 to 28.7)	22.8 (21.2 to 24.5)
85+ (*N* = 172,420)	32.3 (28.2 to 36.6)	34.8 (30.6 to 39.2)	33.2 (30.2 to 36.4)
Total (55+) (*N* = 2,215,637)	17.7 (16.7 to 18.7)	19.2 (18.4 to 20.1)	18.4 (17.8 to 19.1)

Prevalence is given as a percentage of people in that cell. Marginal totals (Ns) are for the population (not the sample) based on Australian Bureau of Statistics data for 2018 [20]. The mean age of men and women within each age category differed by less than 0.2 years

There were moderate associations between prevalence of difficulty using hands, arms or legs and each of the control variables used to obtain sampling weights: the odds ratio (OR) for age 85+ compared to age 55–64 was 2.6 (95% CI 2.2 to 2.9), the OR for women compared to men was 1.23 (95% CI 1.16 to 1.31), and the OR for the geographical area with highest compared to lowest prevalence was 1.9 (95% CI 1.4 to 2.6). Nonetheless, the crude estimates and adjusted estimates of population prevalences of motor impairment were very similar. Adjusted estimates are reported here. The estimated prevalences of muscle weakness, fatigue, contracture, impaired balance and impaired coordination are shown in Table 2.

**Table 2:** Estimated prevalence (%) of motor impairments by age and sex

Age	Men	Women	All
**Weakness**			
55–64	7.1 (6 to 8.3)	11.4 (10.4 to 12.6)	9.3 (8.6 to 10.1)
65–74	8.8 (7.8 to 9.9)	12.3 (11.3 to 13.4)	10.6 (9.9 to 11.3)
75–84	11.7 (9.9 to 13.8)	16.1 (14.3 to 18.1)	14.1 (12.8 to 15.5)
85+	21.2 (17.7 to 25.1)	22.5 (19 to 26.4)	22 (19.4 to 24.8)
Total (55+)	9.3 (8.6 to 10.1)	13.6 (12.9 to 14.4)	11.6 (11 to 12.1)
**Fatigue**			
55–64	5.5 (4.5 to 6.6)	6.2 (5.4 to 7)	5.8 (5.2 to 6.5)
65–74	6.5 (5.7 to 7.5)	8.3 (7.5 to 9.2)	7.4 (6.9 to 8.1)
75–84	10.8 (9.1 to 12.7)	11.6 (10.1 to 13.3)	11.2 (10.1 to 12.4)
85+	18.9 (15.6 to 22.8)	21.6 (18.1 to 25.5)	20.6 (18 to 23.4)
Total (55+)	7.5 (6.9 to 8.2)	9.3 (8.7 to 9.9)	8.5 (8 to 8.9)
**Contracture**			
55–64	7.1 (6.1 to 8.3)	6.3 (5.5 to 7.3)	6.7 (6 to 7.4)
65–74	8 (7 to 9.1)	7.9 (7.1 to 8.8)	8 (7.3 to 8.6)
75–84	9.8 (8.2 to 11.7)	10.2 (8.7 to 11.8)	10 (8.9 to 11.3)
85+	15.2 (12.1 to 18.9)	12.3 (9.7 to 15.4)	13.4 (11.3 to 15.7)
Total (55+)	8.4 (7.7 to 9.1)	8.1 (7.5 to 8.7)	8.2 (7.8 to 8.7)
**Impaired balance**			
55–64	1.7 (1.2 to 2.4)	2.4 (1.9 to 2.9)	2.1 (1.7 to 2.5)
65–74	2.3 (1.8 to 2.8)	3.8 (3.2 to 4.5)	3 (2.7 to 3.5)
75–84	5.4 (4.3 to 6.8)	7.5 (6.3 to 8.9)	6.5 (5.7 to 7.5)
85+	13.7 (10.9 to 17.1)	16.7 (13.6 to 20.3)	15.6 (13.3 to 18.1)
Total (55+)	3.2 (2.8 to 3.7)	5.1 (4.6 to 5.6)	4.2 (3.9 to 4.6)
**Impaired coordination**			
55–64	2.5 (1.9 to 3.3)	2.3 (1.8 to 2.8)	2.4 (2 to 2.8)
65–74	2.8 (2.3 to 3.5)	3.4 (2.8 to 4)	3.1 (2.7 to 3.6)
75–84	3.9 (3 to 5)	5.6 (4.5 to 6.9)	4.8 (4.1 to 5.7)
85+	9.8 (7.5 to 12.7)	7.9 (5.8 to 10.6)	8.6 (7 to 10.5)
Total (55+)	3.3 (2.9 to 3.7)	3.7 (3.3 to 4.2)	3.5 (3.2 to 3.8)
**Any (at least one) of these impairments**			
55–64	13.7 (12.3 to 15.3)	17.6 (16.3 to 19)	15.7 (14.7 to 16.7)
65–74	16.5 (15.2 to 17.9)	21.1 (19.9 to 22.5)	18.8 (17.9 to 19.8)
75–84	23.4 (21.1 to 25.8)	28.5 (26.3 to 30.8)	26.1 (24.5 to 27.8)
85+	37.9 (33.6 to 42.4)	44 (39.7 to 48.5)	41.8 (38.6 to 45)
Total (55+)	17.7 (16.8 to 18.7)	23.2 (22.3 to 24.1)	20.6 (19.9 to 21.3)

Prevalence is given as a percentage of people in that cell

## Discussion

To our knowledge these are the first population-based estimates of the prevalence of specific motor impairments in the Australian population. Unlike most previous population-based studies of motor impairment this study estimates the prevalence of multiple motor impairments. We estimate that, in residents of New South Wales aged 55 and over, the prevalence of specific impairments is as follows: weakness 12%, fatigue 9%, contracture 8%, impaired balance 4% and impaired coordination 4%. The prevalence of having at least one of these impairments is 21%. In the population aged 85 and over, the prevalence of at least one of these impairments is 42%. In comparison, the three most prevalent global diseases (Level 3 Global Burden of Diseases, Injuries, and Risk Factors Study causes) are oral disorders, headache disorders and latent tuberculosis infection, with estimated global prevalences (across all ages and all countries) in 2017 of 46, 40 and 26% respectively (calculated from reference [1] assuming a global population of 7.511 billion in 2017 [21]).

Two clear patterns emerge from the data. First, women consistently experience more difficulty using their hands, arms or legs and have more motor impairments than men. This was particularly evident for difficulty using hands, and for weakness and fatigue. Second, there was a large increase with age in difficulty using hands, arms or legs and in the prevalence of all motor impairments. There was at least twice the prevalence of all motor impairments in the group aged 85+ compared to the group aged 55–64. The prevalence of poor balance in the group aged 85+ was more than seven times that in the group aged 55–64.

Our estimates of the population prevalence of specific motor impairments are generally comparable to or lower than previous estimates. For example, it has been estimated that the population prevalence of weakness is 4–18% at 60–69 years, 9–34% at 70–79 years, and 26–68% at 80+ years [7, 9], and 5–31% at 60+ years [5, 8]. Estimates for fatigue are 38% at 18–45 years [11], 12% at 18+ years [12] and 22% at 19–80 years [10]. Estimates for impaired balance or coordination are 8–14% at 60–69 years, 16–17% at 70–79 years, and 39–46% at 80+ years [14, 15], 16% at 60+ years [13] and 28% at 65+ years [16]. Other studies have also report that women have higher prevalences of impairment [22] and that prevalence of weakness and impaired balance increase markedly with age [7, 9, 14, 15].

One explanation for the moderate variation in reported prevalences is that there were differences in case definitions across studies. Most previous studies of the population prevalence of weakness have defined cases as people with grip strength less than a specific threshold. In those studies grip strength was measured objectively. In the present study, cases or their surrogates self-reported that they had difficulty using their hands, arms or legs to carry out everyday activities because of weakness. While this approach relies on subjective self-reports, it explicitly assesses the weakness of all appendicular muscles (though it does not assess weakness of axial muscles). Previous studies of the population prevalence of specific motor impairments have not explicitly required that the motor impairment limits motor ability, although many studies have demonstrated associations between fatigue and motor ability (e.g., [23]). Studies of the prevalence of fatigue have used definitions of fatigue that reflect both the physical and the emotional or perceptual dimensions of fatigue. Interestingly, a population-based study that explicitly measured different dimensions of fatigue found similar distributions of physical and mental fatigue [22]. The present study asked participants if their “muscles tire easily” causing “difficulty using your hands, arms or legs to carry out everyday activities”. This wording emphasises the physical dimension of fatigue. Several population-based studies have quantified balance impairment using the Short Physical Performance Battery. One study conducted in the USA that used case definitions similar to those used here found that 28% of participants aged 65+ reported “problems with balance or coordination” whereas 4% of our population aged 55+ reported difficulty using hands, arms or legs to carry out everyday activities because of poor coordination and 4% reported poor balance.

The current study has several limitations. First, motor impairments were self-reported or reported by a surrogate. Self- or surrogate-reported presence or absence of a motor impairment may not always correspond with the judgement of a trained observer. This is likely to be particularly problematic for the estimates of contracture because it is likely many respondents including some who had contractures did not clearly understand what was meant by this term. Second, the survey provided data on the prevalence of motor impairments that respondents perceived “caused difficulty using the limbs”. It is likely that the level of “difficulty” experienced by respondents varied from mild to severe. A third limitation is that the response rate was low. Responses were obtained from 36% of the cohort who were invited to provide follow-up data in 2018, and the cohort consisted of the 18% who responded to the initial survey between 2006 and 2009. Thus the data provide potentially biased estimates of prevalence. It was possible to significantly reduce the potential for bias because the sample was obtained from a well-defined and well-characterised population, enabling tight calibration using the known three-way distribution of age, sex and geographical area (224 cells). Nonetheless, there may still be substantial bias if factors other than age, sex and geographical location were strongly associated with both response frequency and the prevalence of motor impairment but not with age, sex or geographical area [24].

This study shows that the prevalence of five specific motor impairments is high in New South Wales residents aged 55 and over. These impairments probably mediate geriatric syndromes [25] that are major causes of morbidity in older populations. Historically, epidemiologists have focused on quantifying the prevalence of diseases and, to a lesser extent, on the prevalence of injuries, risk factors for disease (such as high blood pressure) and non-motor impairments (such as blindness). To the extent that highly prevalent motor impairments share common mechanisms, have similar consequences and respond to the same interventions there may be merit in considering motor impairment as a significant public health problem in its own right.

## Conclusion

The prevalence of specific motor impairments in older Australian adults is high - comparable to that of the most prevalent diseases. In this population, motor impairment is more prevalent in women than men, and increases greatly with age. There may be merit in considering motor impairment as a significant public health problem in its own right.

## Data Availability

The data that support the findings of this study are available from The Sax Institute but restrictions apply to the availability of these data, which were used under license for the current study, and so are not publicly available. Data may be made available upon request to The Sax Institute.

## Abbreviations

ASGS: Australian Statistical Geography Standard
SA4: Statistical area level 4
OR: Odds ratio
